# Disability-Disaggregated Data Collection: Hospital-Based Application of the Washington Group Questions in an Eye Hospital in Paraguay

**DOI:** 10.3390/ijerph16173085

**Published:** 2019-08-25

**Authors:** Manfred Mörchen, Olmedo Zambrano, Alexander Páez, Paola Salgado, Jason Penniecook, Andrea Brandt von Lindau, David Lewis

**Affiliations:** 1 Christoffel Blindenmission (CBM) International, Stubenwaldallee 5, 64625 Bensheim, Germany; 2 Fundaciόn Visiόn, Ingavi, Fernando de la Mora 8000, Paraguay; 3 CBM Australia, 56 Rutland Rd, Melbourne 3128, Australia

**Keywords:** inclusive eye health, disability, health information system, Washington Group

## Abstract

Disability-disaggregated data are increasingly considered important to monitor progress in Universal Eye Health Care. Hospital-based data are still elusive because of the cultural ambiguities of the term disability, especially in under-resourced Health Information Systems in low-and middle-income countries. The aim of this study was to estimate the hospital-based rate of disability in patients presenting at an eye department of a rural hospital in Paraguay and to discuss implications for the management of access barriers. Therefore, we introduced two standardized sets of the Washington Group (WG) Questions as a pilot project. In total, 999 patients answered the self-report WG short set (WG-SS) questionnaire with six functional domains, and 501 of these patients answered an extended set, which included additional domains for “anxiety” and “depression” (WG-ES3). Overall, 27.7% (95% Confidence Interval (CI) 24.9–30.3) were categorized as having a disability. A total of 9.6% (95% CI 7.9–11.6) were categorized as having a disability because of communication difficulties, which was second only to visual difficulties. The odds ratio for disability for patients aged 70 years and older was 8.5 (95% CI 5.0–14.4) and for male patients, it was 0.83 (95% CI 0.62–1.1). Of those patients who answered the WG-ES3, 3.4% were categorized as having a disability because of being worried, nervous or anxious and 1.4% because of feeling depressed. An analysis of the questions of the “depression” domain was impeded by a high rate of measurement errors. The results of the different domains can now be used to inform the identification and mitigation of potential access barriers to eye health services for different types of impairments.

## 1. Introduction

Universal access and equity of eye health services are salient cross-cutting principles of the World Health Organization (WHO)’s global action plan 2014–2019 [1]. The agenda of the Sustainable Development Goals with its principle of `Leaving no one behind’ suggests giving high priority to indicators that focus on hard-to-reach populations. As a prerequisite, health data need to be disaggregated not only by demographic and socioeconomic information but also by characteristics such as disability, ethnicity, migratory status, etc. [2,3,4] People with disabilities are “likely to experience health inequities” [5], but data about their access to eye health services are still elusive [6]. This is concerning because people with disabilities make up a significant proportion of the global population. The monitoring of equity-oriented Universal Health Care is not possible if health systems do not provide more accurate information about people with disabilities accessing health services [7]. Reasons for insufficient disability-disaggregated data collection include under-resourced health systems, cultural ambiguities of understanding disability and its connotations, as well as a lack of awareness about the importance of socio-cultural determinants of health for people with disabilities [8]. 

Disability, including that caused by visual impairment, is closely associated with age and poverty [9]. An estimated 31 million of 36 million blind people globally are aged 50 years and older [10]. Given these associations, eye hospitals especially in low- and middle-income countries often pro-actively target patients from poorer populations, in order to increase their access to eye health. Also, people with other disabling conditions are often more affected by visual problems because of the association of visual impairment and co-morbidities or intersecting disabilities, such as those caused by hearing difficulties, mental illness, etc. [11,12,13]. For example, people with Down syndrome have higher risks of developing co-morbid conditions such as refractive error. However, there are significant barriers impeding the access of people with disabilities to eye health services including physical and financial barriers, communication difficulties and diagnostic overshadowing [14,15]. The results of a survey of adults with registered visual impairment in the UK suggested that intersecting disabilities could compound the communication between eye health professionals and patients with unavoidable visual impairment “… as many also have health and/or communication difficulties in addition to their visual impairment (70% reporting this to be the case, and many reported hearing difficulties).” [13]. Barriers related to the way health professionals do their work are common as well. According to Lagu, “[t]he greatest challenge in achieving universal accessibility in health care settings may not be the cost but the need for a change in mindset on the part of clinicians and administrators” [16]. It has also been reported that health care staff tend to underestimate the need for comprehensive accessibility, in that they neglect aspects beyond physical accessibility. Sanchez pointed out that “‘[p]erceived accessibility’ appears to be based solely on basic physical access, without consideration of the real needs and issues of persons with mobility impairments.” [17].

It is, therefore, important for an eye hospital to know whether people with disabilities are accessing their services and, if so, to what extent. Disability-disaggregated data could inform eye health staff if more needs to be done to improve the equitability of their services. For example, a low number of patients with hearing difficulties could indicate the presence of communication barriers in the eye hospital. Furthermore, these barriers may also exist within the referral networks of that hospital. All potential barriers should be investigated to inform mitigation strategies [18]. The association of visual or multiple impairments with a “comorbidity of socioeconomic deprivation” [19] needs to be taken into account throughout the management of eye health services. A national analysis of administrative disability-disaggregated data collected from different eye hospitals could indicate the level of equity of eye health services, and triangulation with data from population-based surveys could suggest the extent to which Tudor Hart´s inverse care law (“the availability of good medical care tends to vary inversely with the need for the population it served”) is still relevant [20]. 

There are a few examples of countries and eye health programs that are moving towards a greater focus on equity in eye health planning, including specific consideration of people with disabilities [21,22]. For example, the next Kenyan national eye health plan 2019–2023 will trial “inequality monitoring in eye departments (…) to determine the feasibility of expanding the social variables collected (e.g., socioeconomic status, place of residence, disability and social support)” [23]. However, feasible approaches for collecting disability-disaggregated data in countries with underdeveloped health systems are elusive.

One of the available tools—the ‘Washington Group (WG) Short Set (SS) of questions’—has already been used in population-based surveys and censuses [24]. The underlying conceptual framework for this tool is the ‘International Classification of Functioning, Disability, and Health’ (ICF), which presents a bio-psychosocial model. Rather than mainly assessing body functions and clinical impairments, the WG questions focus on ‘activity limitation’ as one of the main components of the ICF [25,26]. The questions aim to identify people with self-reported difficulties in functioning in six universal actions that put them at risk of restricted participation in an unaccommodating environment. The WG short set (WG-SS) questions were “designed to provide comparable data cross-nationally for populations living in a variety of cultures with varying economic resources” [26]. Recently, programs in various settings have started to use the WG-SS beyond population-based surveys to determine whether people with disabilities access their services or to inform aspects of equality in service delivery [27]. There are also first reports of the application of the WG-SS in eye hospitals and related programs [28,29,30]. 

Actual disability-related data for Paraguay are elusive. The World Report on Disability suggested an overall age-standardized disability prevalence of 10.4% among adults aged 18 years and over in the year 2002 [9], and Mitra et al. reported a national prevalence of 9.6% [31]. The 2013 shadow report of the United Nations Convention on the Rights of Persons with Disabilities estimated a national prevalence rate of 12% [32]. The report also highlighted differential accessibility barriers, such as waiving public transportation fees exclusively for people with visual impairment but not for people with other impairments. An association between age and psycho-social impairments was reported in the World Health Survey Report Paraguay: 6.0% of participants of the age group 18–29 reported severe or extreme problems with “feeling sad, low or depressed”, and this increased to 9.6% and 20.9% for the age groups 70–79 and 80+, respectively [33]. 

In a similar way to many other cultures, it is difficult to translate the meaning of “disability” into the widely used local language Guarani, because it “lacks a term that could encapsulate the real meaning of the expression ‘person with a disability’” [32]. To mitigate these language barriers, it is therefore important to use data collection tools that avoid the use of the term ‘disability’, such as the WG-SS.

The overall objective of this study was to pilot a method for counting how many patients with self-reported impairments attended an eye health hospital in rural Paraguay. The pilot tested the introduction of two versions of the WG questions. The first version (short set WG-SS) was employed to assess the rate of patients with self-reported difficulties in six domains, with the aim of estimating the age- and sex-disaggregated rate of people with disabilities accessing the hospital. The second version (WG extended set (WG-ES 3)) assessed the additional rate of patients with self-reported difficulties in the “anxiety” and “depression” domains, and aimed to estimate the rate of patients with psycho-social disability. The results were used to discuss practical implications in regard to access barriers to eye health services for patients with disabilities in Paraguay.

## 2. Materials and Methods

Data collection was undertaken from 10 July 2018 to 07 September 2018 at Clinica Belen, a busy rural satellite eye hospital of the tertiary eye hospital Fundaciόn Visiόn (FV), in the upper middle-income country Paraguay. Clinica Belen is located in Coronel Oviedo, approximately 120 km west of the capital Asunciόn. Its catchment area consists of an estimated one million people from three provinces (Caaguazu, Guaira and Caazapa). With 10 to 60 people per km^2^, the area is one of the more densely populated regions in Paraguay (national average 12.7 per km^2^) [34]. The hospital serves approximately 70 patients per day and offers cataract, glaucoma, corneal and retinal eye health services. The hospital collaborates with and refers patients to community-based inclusive development services and implements low vision self-help groups for patients with unavoidable visual impairment. A basic Health Management Information System (HMIS) exists which collates data on basic parameters such as sex, medical diagnosis, etc. It was assumed that the hospital staff were able to apply the WG questions without additional financial or human resources. 

The original English WG questions were translated into Paraguayan Spanish and then into the main target language Guarani by team members of FV hospital (native Paraguayan Spanish and Guarani, with English as a secondary language), employing a conceptual translation method (A.P., J.P., P.S., and O.Z.) [35]. The translation strived to convey the meaning of the WG questions in the cultural context of Paraguay. The resulting translations are presented in Table 1 and Table 2.

Two WG questionnaires were selected as data collecting tools:

1. The WG Short Set (WG-SS) consists of a set of six single short questions on functional domains (which are seeing, hearing, walking or climbing stairs, remembering or concentrating, self-care, and communication). The questions were designed for applications in populations aged five years and older and will identify the large majority of people with disabilities. The answer options are “no difficulty”, “yes, some difficulty”, “yes, a lot of difficulty”, and “cannot do at all” (response pattern 1 according to the WG guidelines) [36].
2. The WG Extended Set on Functioning (WG-ES 3) consists of the same questions as the WG-SS and adds four additional questions related to two domains of “affect” (anxiety and depression) for participants aged 13 years and older. The answer options regarding the frequency of feeling anxious or depressed are “daily”, “weekly”, monthly”, “a few times a year”, and “never”. The severity can be answered with “a little”, “a lot” and “somewhere between a little and a lot” (response patterns 5 and 6 according to WG guidelines) [36]. We excluded the “upper body” questions of the WG-ES 3 in order to improve the feasibility of the data collection [36].

The decision to also test the WG-ES 3 was informed by the assumption that age, visual and psycho-social impairments are closely linked. We assumed that there might be significant barriers for this patient group in Paraguay, considering the estimated national prevalence of blindness of 1.1% (95% CI 0.6–1.6) for people aged 50 years and older, a predominantly older population accessing the eye hospital, a high national prevalence of older people with psycho-social impairments and underdeveloped community-based mental health services [37,38]. 

Because of limited time and budget constraints, it was not possible to conduct a cognitive interview testing of the translated questions [39]. The translated questions were tested with patients over two days, and the interviewer did not encounter any challenges regarding the WG-SS. The term “depressed” used in questions 9 and 10 (WG-ES 3), was very difficult to translate into Guarani, and it was at times necessary to provide additional explanations to the respondents. An on-site training session and workshop for the data collecting staff of Clinica Belen was conducted by advisors (P.S. and O.Z.) with extensive experience in Community-Based Inclusive Development in Paraguay (2 days at FV, 4 days at Clinica Belen). The training also covered aspects of disability-inclusive development. After the training, the WG questions and templates were tested for two days before data collection commenced. Data collecting staff were selected from the existing staff of Clinica Belen (social workers responsible for visual acuity tests and pharmacy, and administrative assistants) with no additional staff employed. Data were entered manually in separate paper forms which were attached to the patients` records. The data were then entered into an Excel spreadsheet by hospital data clerks and uploaded into the HMIS of FV hospital on a daily basis. Cleaning and analysis of the data were done by FV staff (A.P., J.P.) at the end of each day (or as possible based on the availability of internet connection). The short and extended versions of the questionnaire were used in alternating weeks. The questionnaire was applied only once per patient during the pilot phase in order to avoid double-counting of patients who presented again for any follow-up visit. On-site checking of the processes was conducted randomly by FV staff (A.P.).

### 2.1. Data Analysis WG-SS

We followed the categorization for disability as suggested by the Washington Group on Disability Statistics: “ …the level of inclusion in at least one domain/question is coded [a lot of difficulty] or [cannot do at all].” [36] Data entered on an Excel spreadsheet were summarized descriptively by calculating mean, median and inter-quartile range for male and female participants. Frequency tables for each of the six domains and disability were generated as well as for the age categories 5–19, 20–49, 50–69 and ≥ 70 years. The rate for each domain and disability was calculated as percentages with 95% Confidence Intervals (applying Clopper–Pearson single proportion test). Odds Ratios (OR) with exact Fisher 95% confidence intervals (CI) were calculated to estimate the risk of reporting “a lot of difficulties” or “cannot do at all” in all six domains and then being categorized as a person with disability, disaggregated by sex. Univariate logistic regression analysis was conducted to estimate the same risks of reporting by generating ORs and 95% CIs for the different age groups. The results were adjusted for sex as a possible confounding factor, and multivariate logistic regression was conducted to generate adjusted OR and 95% CIs. All tests were conducted with StatsDirect (version 3.1.22) (StatsDirect LTD, Birkenhead, United Kingdom).

### 2.2. Data Analysis WG-ES 3

We followed WG-ES 3 exactly for the calculation of frequencies for the two categories of the domain “affect”, resulting in four indicator levels: “the level of inclusion […] for the domains Anxiety, Depression, […] the highest level of difficulties on a four-point scale.” [36]. The level four indicator included those patients who reported feeling ‘worried, nervous or anxious’ and/or ‘depressed’, at a frequency of ‘daily’, and with a severity of ‘a lot’. These patients were then categorized as having psycho-social disability. Cross-tabulations were built according the WG recommendations. The tabulations combined the results for the questions regarding frequency and severity of the domains and resulted in the four indicator levels. Percentages were calculated for each of the indicators, disaggregated by sex.

All subjects gave informed consent before they participated in the study. The study was conducted in accordance with the Declaration of Helsinki, and the protocol was approved in May 2018 by the Institutional Review Board of Fundación Visión Hospital.

## 3. Results

A total of 1021 patients were asked to answer the WG questionnaires. Over 90% of the patients answered the Guarani version of the questions. A total of 22 questionnaire responses were not included in the analysis as they were erroneously conducted with parents of children aged less than five years. This resulted in 999 (male: 443 = 44.3%; female: 556 = 55.7%) patients whose questionnaires were analyzed, with 498 (49.8%) patients having answered the short version and 501 (50.2) having answered the extended version.

**Figure 1:**
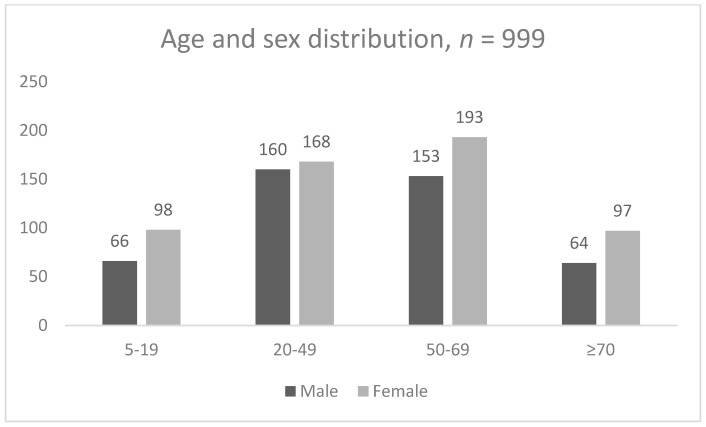
Age and sex distribution of participating patients.

The mean age of the patients was 47.6 years (male = 47.3; female = 47.8 years), and the age and sex distribution is shown in Figure 1. The median age was 51 years (male = 50.0; female = 52.0 years) with an inter-quartile range of 37 years (28–65 years; male = 29–64; female = 26–66 years).

### 3.1. Short Set WG-SS

Overall, 27.7% (95% CI 24.9–30.3) of the patients reported ‘a lot of difficulties’ or ‘cannot do at all’ in at least one domain, and they were categorized as patients with disability. The most common domain was ‘visual’ at 16.4 % (14.2–18.9), followed by ‘communicating’ at 9.6% (7.9–11.6), ‘remembering’ at 5.9% (4.5–7.6), ‘mobility’ at 3.8% (2.7–5.2), ‘hearing’ at 3.5% (2.5–4.8), and ‘self-care’ at 1.9% (1.1–3.0). The results are summarized in Table 3.

The number of patients reporting ‘some difficulties’ was much higher than those reporting ‘cannot do at all’ or ‘a lot of difficulties’. For example, having some difficulties with hearing was reported by 10%, compared to 1% reporting ‘cannot do at all’; and particularly having ‘some difficulties’ with remembering was reported by 33.1%, compared to only 0.9% reporting ‘cannot do at all’. The difference in the rate of those reporting ‘some difficulties’ versus ‘cannot do at all’ was not large in the self-care domain (2.1 versus 1.6%).

The rate of disability between the different age groups suggest much higher rates in the oldest age groups. Overall, 61.5% (53.5–69.0) of all patients aged ≥ 70 years were categorized as patients with disability, compared to 14.6% (11.0–19.0) of the age group 20 to 49 years. Self-care was the domain with the smallest difference between the age groups. The OR of the oldest age group to report any type of disability was 8.5 (5.0–14.4). The results are summarized in Table 4.

A total of 32% of those categorized as having a disability had reported difficulties in two or more domains (for example, 5% reported difficulties in three domains).

### 3.2. Extended Set WG-ES 3

#### 3.2.1. Affect/Anxiety Domain

A total of 501 patients answered the WG-ES 3 questionnaire (male = 223; female = 278), and 477 answers were included in the analysis of the affect/anxiety domain. A total of 24 patients (4.8%) were excluded due to a variety of reasons (refused to answer = 4, patients do not know = 10, and error = 10). The results of the cross-tabulation of the domain are shown in Table 5 and Table 6. A total of 18 patients (3.6%; male: 1 = 0.4%; female: 17 = 6.1%) answered that they felt worried, nervous or anxious a lot on a daily basis (level 4); they were categorized as patients with disability.

#### 3.2.2. Affect/Depression Domain:

A high number of 151 patients (30.1%) had to be excluded due to a variety of reasons (refused to answer = 3, patients do not know = 7, and error = 141). The high number of errors resulted mainly from the fact that patients indicated a level of depression even when answering that they never feel depressed. The results of the cross-tabulation of the affect/depression domain are shown in Table 7 and Table 8. Overall, seven patients (1.4%; male: 1 = 0.4%, female: 6 = 2.2%) answered that they felt depressed a lot on a daily basis (level 4); they were categorized as having a disability.

## 4. Discussion

To our knowledge, this is the first report of the application of two sets of the WG questionnaires in an eye hospital in a middle-income country. Over two months, a total of 999 patients answered the WG-SS questionnaire, and 501 of these patients answered the extended set (WG-ES 3). Overall, 27.7% (95% CI 24.9–30.3) were categorized as having a disability because of self-reported severe difficulties in at least one domain, and the oldest age group had much higher odds of reporting severe difficulties (OR = 8.5 (95% CI 5.0–14.4)). The OR for male patients to be categorized as having a disability was 0.83 (95% CI 0.62–1.1). Additionally, 3.6% of the patients who answered the extended set WG-ES 3 were categorized as having a psycho-social disability in the anxiety domain, and this was 1.4% in the depression domain.

The overall rate of disability was higher compared to results from an eye care program that tested the WG-SS questions with a large number of patients in an urban low income settlement in Bhopal, India [30]. The Indian results suggested a combined rate of disability of 16.7% (CI not reported). The results for older patients were similar to Paraguay (OR for patients being 50 years and older = 3.5, *p* < 0.001). Female patients in India had higher odds of reporting severe difficulties (OR 1.4, *p* < 0.0001). The percentage of patients with a lot of difficulties or inability in two or more domains at 28% was very similar to the result from Paraguay [30]. 

### 4.1. WG-SS Results

#### 4.1.1. Vision Domain

The percentage of people reporting having ‘a lot of difficulties’ or ‘cannot do at all’ was highest in the vision domain, which is to be expected for a population presenting at an eye hospital. The association between age and visual impairment was also confirmed, with 36.7% (95% CI 29.2–44.6%) of the age group ≥ 70 years being categorized as having a disability because of visual difficulties. The possibility of an overestimation because of false-positive answers in this domain should be kept in mind. The glasses clause of the WG question of the domain vision (“even when wearing glasses”) was reported to be one of the main reasons for possible false-positive answers [40]. The results of a previous cognitive testing of the WG-SS in Paraguay suggested a high percentage of ‘glasses wearers’ who misunderstood the glasses clause (40.5%), and provided false-positive answers [40]. 

#### 4.1.2. Communicating Domain

The communicating domain was at 9.6% (95% CI 7.9–11.6)—the second most common domain in which patients reported significant limitations (‘a lot of difficulties’ or ‘cannot do at all’), especially for the oldest age group (OR = 7.9 (95% CI 3.2–19.3)). This is an important finding in the context of Paraguay. The results of the World Health Survey Paraguay indicated that communication with health staff was generally perceived as being important: 38.7% of respondents reported that it is extremely important, with communication ranked higher compared to domains such as ‘choice of health care provider’ (28.8%), ‘autonomy’ (27.7%) or ‘social support’ (32.4%) [33]. At the same time, 11.8% of the respondents assessed the ‘time for questions’ for hospital inpatient services in Paraguay as ‘moderate’, ‘bad’ or ‘very bad.’ This was especially an issue for poorer patients (quintile 2 = 13.9% versus quintile five = 8.8%) [33]. High numbers, especially of patients with long-term visual impairment and self-reported communication difficulties, were also reported by other studies [13]. Communication barriers can be an important cause of poorer medical outcomes, resulting in increased health disparities for people with disabilities [41]. The results of the WG questions could be used to further analyze how the staff at Clinica Belen communicates, especially with those reporting a lot of communication difficulties, and whether training is needed for staff to develop the skills needed to “engage in open and effective communication” [42] with patients with disabilities.

#### 4.1.3. Remembering/Concentrating Domain

Improved communication will also be important to assist patients who reported severe difficulties with remembering (5.9% 95% (CI 4.5–7.6)). Male patients had lower odds of reporting severe difficulties with remembering or concentrating (OR = 0.41 (95% CI = 0.21–0.76)), which was similar to results of the World Health Survey Paraguay (2.3% of male respondents reported severe or extreme difficulties remembering or concentrating in the last 30 days, compared to 5.9% of female respondents) [33]. These results should have practical consequences. For example, patients with severe difficulties remembering might need additional support with medical adherence especially for those eye diseases which require long-term treatment. Also, barriers to medical information for patients with cognitive impairments need to be identified, and especially written information such as consent forms, discharge instructions, etc., should be readily available in easy to read formats [16,43]. 

#### 4.1.4. Self-Care Domain

Overall only 1.6% of the patients reported ‘a lot of difficulties’ or ‘cannot do at all’ in the self-care domain. This was still more than results from the World Health Survey Paraguay, especially for older patients: 2.4% of people aged 60 to 69 years in Paraguay reported severe or extreme difficulties with self-care in the last 30 days—whereas in the eye hospital results, 6.2% (95% CI 3.0–11.1) of the patients in the oldest age group reported severe difficulties [33]. The Washington Group on Disability Statistics recommended that this question might be omitted in countries where cultural barriers constituted questions about self-care as inappropriate [26]. For example, it was not possible to introduce a ‘self-care’ question at an eye hospital in Cambodia that applied a modified version of the WG questions [28]. It was also reported that false-negative responses resulting in underestimation in this domain were less problematic than with other domains. Miller et al. suggested that 96.8% of those providing false-negative responses in the domain self-care were still very likely to be categorized as persons with disabilities because of simultaneous reporting of severe difficulties in other domains [40]. However, the inability to perform specific Activities of Daily Living (ADL), such as washing and dressing, might be a risk factor for increasing the barriers to accessing eye health services. For example, Friedman et al. reported that highly dependent nursing home residents with diagnosed cataract who are likely to need substantial support in ADLs face significant barriers to accessing cataract surgery: “Without a program to assist residents in identifying a surgeon, making it to the appointment, and getting to the hospital for surgery, only 2 (2%) of 99 identified by an ophthalmologist as having decreased vision due to cataract received surgery.” [44]. Also, family members or guardians of highly dependent patients often do not give consent to cataract surgery, for example, which might be one possible explanation for the overall low rate of patients with reported difficulties who access the eye hospital [44]. Therefore, we deem it important to apply the WG question regarding self-care, if culturally appropriate, as it may be an important indicator for patients who face substantial barriers to accessing eye health services.

#### 4.1.5. Mobility Domain

Significant limitations (‘a lot of difficulties’ or ‘cannot do at all’) in the mobility domain were less frequently reported in Paraguay. They were more commonly reported in the oldest age group (14.9% (95% CI 9.8–21.4)). The overall results can be corroborated by results from the World Health Survey Paraguay (severe or extreme difficulties with moving around in the last 30 days were reported by 1.3% of men, 3.2% of women, and 20.6% of the age group 80+) [33]. It is important to remove physical barriers for this patient group as much as possible, including barriers to accessing medical equipment. It was already reported that patients with mobility difficulties have significant difficulties accessing eye health services, even in high-income countries: “Among practices that might not require transfer, ophthalmology had the highest number of inaccessible practices (8[25%]).” [45] Also, accessibility of medical equipment seems to be a far more common barrier than accessibility of buildings. It needs to be verified whether patients reporting difficulties with mobility are able to conveniently use ophthalmological equipment such as slitlamps, operating theatre (OT) tables, etc. [16]. 

#### 4.1.6. Hearing Domain

The oldest age group in Paraguay had higher odds of reporting severe hearing difficulties (OR = 10.8 (95% CI 2.5–47.3)), which is not unexpected when considering the association of age and hearing difficulties. However, the overall percentage of patients being categorized as having a disability because of hearing difficulties was only 3.5% (95%CI 2.5–4.8). Considering results from population-based studies, this does not seem to be high. For example, data from India using the WG questions in a cross-sectional study suggested that 25% of those people with visual impairment also had moderate or severe hearing difficulties [11]. Also, a survey amongst adults registered with visual impairment in the UK suggested that “43% reported having hearing difficulties” [13]. It will be important to make sure that access barriers for people with hearing difficulties are mitigated as much as possible. The identification of patients with hearing difficulties is also crucial for the medical management of eye diseases. For example, it was reported that eye patients with hearing difficulties might be more prone to low adherence to medical treatment [13,46]. Also, deaf patients using sign language and having eye diseases that affect not only the visual acuity but also the visual field (for example, advanced glaucoma or Usher syndrome) could have substantial difficulties recognizing sign language. This might discourage patients from seeking to access eye hospitals that do not accommodate their needs. Unfortunately, those difficulties are often overlooked. “A low-vision practitioner may more often than not neglect even to ask a patient whether he or she has hearing or communication problems.” [47] The WG questions and accessibility audits focusing on barriers for patients with hearing difficulties in the eye hospital could help to improve access and communication [48]. 

### 4.2. WG-ES3 Results

The results of the four questions of the WG-ES3 need to be interpreted more cautiously, especially because of the observed measurement errors regarding the depression domain. Overall, 30.1% of the answers of this domain were excluded, mainly because of discrepancies that might indicate false-positive answer patterns (for example, patients continued to answer question 10 although they indicated no problem with feeling depressed in question 9). The overall rate of patients being categorized as having a disability because of depression was much lower than that of the World Health Survey Paraguay (1.4%, male = 0.4%, female = 2.2%; compared to 7.1%, male = 4.1, female = 10.1%) [33]. The answers to questions of the anxiety domain were much more consistent, and only 4.8% of responses had to be excluded. The results were again lower than those of the World Health Survey Paraguay (3.6%, male = 0.4%, female = 6.1%, compared to 12.2%, male = 8.3%, female = 16.1%) [33]. Apart from methodological issues of the WG-ES3 application at Clinica Belen—for example insufficient sample size—it should still be ascertained whether there were potential access barriers for patients with psycho-social disabilities. For example, longitudinal cohort studies suggest causal relationships between visual impairment and depression [12]. Eye health staff, therefore, need to be very aware of mental health-related barriers as stigma and exclusion in communities may reduce the likelihood of successful referrals [12]. In particular, patients with dual sensory loss have a higher risk of depression which could have an impact on communication barriers but also increase barriers in other important areas such as accessing transport [46]. It would also be important to evaluate whether diagnostic overshadowing results in fewer patients with mental health difficulties being sent to the eye hospital (for example, primary health staff might view visual impairment as intrinsic to mental health problems and not recommend eye examinations) [49]. 

### 4.3. The WG Questions in a Hospital Setting

Generating knowledge of the number of patients with disabilities accessing hospitals including eye hospitals is an important first step towards creating more equitable health services. Generally, institution-based administrative data cannot be used to estimate prevalence and is considered as subordinate to population-based data in regard to its potential to monitor inequality: “… institution-based data may not be helpful in monitoring equity, unless the social and economic characteristics of the service users can be compared with those of the general catchment population.” [2,50]. The lack of actual population-based data regarding people with disabilities in Paraguay makes it difficult to estimate the denominator of all people with disabilities who might be in need of eye health services. As a consequence, it is not possible to hypothesize whether there were for example more community-based accessibility barriers for people with walking difficulties in Paraguay, which would result in fewer people reporting at the hospital. It was also reported that people with disabilities might prefer eye health services in the vicinity of their communities instead of accessing higher level eye hospitals. “In India people attending OCs (outreach camps) and VCs (vision centers) were much more likely to report functional limitations and self-identify as `disabled’ than people attending the hospital.” [31].

However, lack of population-based data or lack of epidemiological knowledge to interpret available data should not justify insufficient efforts of hospitals to be more engaged in reducing unequal access to their services. Even programs striving to improve access for marginalized groups, including people with disabilities, struggle to demonstrate impact because of elusive quantitative data [22]. Matheson (2018) pointed out that “hospitals hold an inordinate share of power, resources, and influence within health and community systems” [5], and this power should be used much more to “improve their knowledge of local populations with more appropriate data and information, including ways to ensure accountability on equity.” [5].

Ideally, the WG-SS questions would be fully integrated into the Health Information System of an eye hospital in order to use disaggregated data for an equity-orientated analysis of other pertinent indicators, for example cataract surgical outcome monitoring, patient-reported outcome measures or satisfaction surveys. The questions would also be continuously asked of every individual patient, thereby increasing the potential for person-centered care of each of those individuals, based on their self-reported difficulties.

In hospital settings with limited administrative and human resources, it might be possible to reduce the number of questions to an essential set of four questions, with the ‘communicating’ and ‘self-care’ domains excluded, or it may also be possible to remove the glasses and hearing device clauses, to reduce potential confusion for the patient [26]. However, the high number of patients reporting for example communication difficulties at Clinica Belen would not have been identified if we had applied an essential questions-only set. It has also been suggested to apply faster dichotomous answer options, e.g., with yes/no options for disability questions in hospitals, but this might underestimate the rate further and would lead to results which are not comparable with other settings using the WG questions [18,28]. 

### 4.4. Strengths

Both WG questions sets were introduced as recommended by the Washington Group on Disability Statistics, with minor adjustments only. Also, the data analysis followed the WG recommendations, which resulted in data that could be compared with data from other hospitals in rural settings of middle-income countries. In order to improve the external validity of the study, we conducted the research in a clearly defined setting with parsimonious additional financial and human resources.

### 4.5. Limitations

It was not possible to conduct a cognitive interview testing of the translated WG questions, which is a limitation of the study [39]. We tried to mitigate by testing the translated versions with the local population and employing a conceptual translation method. Furthermore, we did not analyze whether there were differences between the small number of patients who answered the Spanish version and those who used the Guarani version. The calculation of the sample size was determined by pragmatic considerations regarding the feasibility of the pilot in a busy secondary-level eye department, without investment in additional human and financial resources. We also did not have prevalence data from studies in comparable settings, especially regarding the WG-ES3 questions, which could have served as a basis for sample size calculation. Therefore, we cannot rule out that the sample size was too small to estimate especially the number of patients with psycho-social disabilities and to provide reliable estimates of the OR, resulting in narrower confidence intervals. Measurement errors regarding the questions of the WG-ES 3 are likely and need to be further examined in future research. Additionally, the incomplete integration of the pilot into the HMIS meant that it was not possible to control for additional covariates of disability, such as income level, quality of residence, ethnicity, etc.

## 5. Conclusions

Overall, 27.7% (95% CI 24.9–30.3) of all patients who accessed the rural eye hospital in Paraguay were categorized as having a disability because of self-reported difficulties in the six domains of the WG-SS. The extended set WG-ES 3 identified 3.6% of patients with psychosocial disabilities in the ‘anxiety’ domain, and 1.4% in the ‘depression’ domain. The results could have several implications for the hospital: domains with a high number of patients reporting (for example, communication difficulties) could lead to implementation of strategies improving the hospital-based management of these patients; domains with a low number of patients reporting (for example, self-care) could lead to further collaboration with community-based services in order to mitigate community-based barriers. Implementing the extended set of the WG questionnaires requires additional training of the hospital staff, and the low rate of patients reporting should not result in premature conclusions. If the daily routine application of the WG questions is not feasible, the periodic application of the WG questions can be considered. This could enable the hospital management to monitor changes over time (for example, do more patients with hearing difficulties access the hospital after training of the hospital staff in communicating with patients with hearing difficulties?). Additional research is needed to examine how the WG questionnaires can be used to monitor aspects related to equity (for example, access to cataract surgeries and outcomes for patients with disabilities compared to those without disabilities).

## Figures and Tables

**Table 1:** Translated Washington Group short set (WG-SS) questions and answers into Paraguayan Spanish and Guarani.

	English	Paraguayan Spanish	Guarani
1	Do you have difficulty seeing, even if wearing glasses? a. No, no difficulty b. Yes, some difficulty c. Yes, a lot of difficulty d. Cannot do it at all	¿Ves con dificultad incluso usando lentes? a. No. Veo bien. b. Si. A veces tengo dificultad. c. Si. Tengo mucha dificultad. d. No veo nada.	¿Rehecha asy piko reipururamo jepe lente? a. Nahaniri, ahecha porä b. Heë, sapy’a py’a c. Heë, aguereko hetá. d. Ndahechaiete voi
2	Do you have difficulty hearing, even if using a hearing aid? a. No, no difficulty b. Yes, some difficulty c. Yes, a lot of difficulty d. Cannot do it at all	¿No escuchas bien, inclusive usando audífonos? a. No. Escucho bien. b. Si. A veces tengo dificultad. c. Si. Tengo mucha dificultad. d. No escucho para nada.	¿Nerehendu poraipa reipururamo jepe audífono? a. Nahaniri, ahendua porä b. Heë, sapy’a py’a c. Heë, aguereko hetá. d. Nahenduiete voi
3	Do you have difficulty walking or climbing steps? a. No, no difficulty b. Yes, some difficulty c. Yes, a lot of difficulty d. Cannot do it at all	¿Caminas con dificultad o subes escaleras con dificultad? a. No. Camino bien. b. Si. A veces tengo dificultad. c. Si. Tengo mucha dificultad. d. No puedo caminar.	¿Reguatá asy pa o ejupi asy escalerape? a. Nahaniri, aguatá’ porä b. Heë, sapy’a py’a c. Heë, aguereko hetá. d. Ndaikatuiete aguata o ejupi escalerape
4	Do you have difficulty remembering or concentrating? a. No, no difficulty b. Yes, some difficulty c. Yes, a lot of difficulty d. Cannot do it at all	¿Tiene dificultad para aprender o para recordar? a. No, mi inteligencia está bien. b. Si. A veces tengo dificultad. c. Si. Tengo mucha dificultad. d. No puedo aprender o recordar.	¿Hasy iko ndeve Eaprende haguá, nemandu’a, haguá? a. Nahaniri, che Ivale. b. Heë, sapy’a py’a c. Heë, aguereko hetá. d. Ndaikatuietevoi o aprendé
5	Do you have difficulty (with self-care such as) washing all over or dressing? a. No, no difficulty b. Yes, some difficulty c. Yes, a lot of difficulty d. Cannot do it at all	¿Es difícil para vos limpiarte y vestirte solo? a. No. You puedo solo. b. Si. A veces tengo dificultad. c. Si. Tengo mucha dificultad. d. No puedo hacer nada, necesito a alguien que me ayude.	¿Hasy iko ndeve eñemopoti ha eñemonde haguá ne añó? a. Nahaniri, che ikatu che añó. b. Heë, sapy’a py’a c. Heë, aguereko hetá. d. Ndoikatuiete ajapo aikoteve che ayúda va’eräre
6	Using your usual (customary) language, do you have difficulty communicating, for example understanding or being understood? a. No, no difficulty b. Yes, some difficulty c. Yes, a lot of difficulty d. Cannot do it at all	¿Es difícil para usted hablar o entender a los demás, en nuestro idioma castellano/guaraní? a. No. Capto y hablo bien. b. Si. A veces tengo dificultad. c. Si. Tengo mucha dificultad. d. Poco entiendo, no puedo hablar bien.	¿Hasy iko ndeve eñemongeta haguá ha entende haguá oñeéva nedivepe, ha ña nde ñe’e tepe guaraní/castellano? a. Nahaniri. A pillá pora, añe’e porä b. Heë, sapy’a py’a c. Heë, aguereko hetá. d. Sa’i entende. Ndaikatuí añeë pova

**Table 2:** Translated Washington Group extended set (WG-ES 3) questions and answers into Paraguayan Spanish and Guarani.

	English	Paraguayan Spanish	Guarani
7	How often do you feel worried, nervous, or anxious? a. Never b. A few times a year c. Monthly d. Weekly e. Daily	¿En qué momento te sentís muy preocupado, enojado o ansioso? a. Nunca b. De vez en cuando en el año c. De vez en cuando cada mes d. De vez en cuando cada semana e. A diario	Mba’eicha jave eñeñandú ejepy’apy, o nde pochy o nde py’a tarová? a. Mba’eicha vevo b. Sapy’a py’a Ary c. Sapy’a py’a Ary jasykuéra d. Sapy’a py’a Arapoköindy rehegua e. Tapiaite
8	Level of feelings when you last felt worried, nervous or anxious? a. A little b. Somewhere in between a little and a lot c. A lot	Cuando te sentías preocupado, enojado o ansioso. ¿Cómo te sentías? a. Muy poco preocupado, enojado o ansioso. b. Poco o mucho c. Muchísimo	Pe reï ramo guare ejepy’apy, o nde pochy o nde py’a tarová. ¿mba’eicha eñeñandu? a. Michimimi ejepy’apy, pochy o py’a tarová b. Michimi o Heta avei c. Heta iterei
9	How often do you feel depressed? a. Never b. A few times a year c. Monthly d. Weekly e. Daily	¿En qué momento te sentís demasiado o muy deprimido? a. Nunca b. De vez en cuando en el año c. De vez en cuando cada mes d. De vez en cuando cada semana e. A diario	¿Mba’eicha jave eñeñandú ñembyasyeterei? a. Mba’eicha vevo b. Sapy’a py’a Ary c. Sapy’a py’a Ary jasykuéra d. Sapy’a py’a Arapoköindy rehegua e. Tapiaite
10	How depressed you felt when last time you were depressed? a. A little b. Somewhere in between a little and a lot c. A lot	Pensando en la última vez que se sintió deprimido, ¿qué tan deprimido se sintió? a. Muy poco deprimido. b. Poco o mucho c. Muchísimo	Pe reï ramo guare ñembyasyeterei? ¿mba’eicha eñeñandu? a. Michimimi ejepy’apy, pochy o py’a tarová b. Michimi o Heta avei c. Heta iterei

**Table 3:** Overall and sex-disaggregated rates of six Washington Group functions and of disability.

		Male (*n* = 443)		Female (*n* = 556)		Total (*n* = 999)		
	Domain	*N*	% (95% CI)	*N*	% (95% CI)	*N*	% (95% CI)	OR (95%CI)
1	Visual	67	15.1 (11.9–18.8)	97	17.4 (14.4–20.9)	164	16.4 (14.2–18.9)	0.85 (0.6–1.2)
2	Hearing	14	3.2 (1.7–5.2)	21	3.8 (2.4–5.7)	35	3.5 (2.5–4.8)	0.84 (0.39–1.75)
3	Mobility	15	3.4 (1.9–5.3)	23	4.1 (2.6–6.1)	38	3.8 (2.7–5.2)	0.82 (0.39–1.65)
4	Remembering/Concentrating	15	3.4 (1.9–5.5)	44	7.9 (5.8–10.5)	59	5.9 (4.5–7.6)	0.41 (0.21–0.76)
5	Self-care	8	1.8 (0.8–3.5)	11	1.4 (0.6–2.8)	19	1.9 (1.1–3.0)	0.91 (0.32–2.51)
6	Communicating	45	10.2 (7.5–13.4)	51	9.2 (7.0–11.9)	96	9.6 (7.9–11.6)	1.2 (0.72–1.74)
	Disability	113	25.5 (21.5–30.0)	163	29.3 (25.6–33.3)	276	27.7 (24.9–30.3)	0.83 (0.62–1.1)

Domain 1–6: number and % of respondents reporting “a lot of difficulty” or “cannot do at all”. Disability: number and % of respondents reporting “a lot of difficulty” or “cannot do at all” in at least one of the six core domains. OR (odds ratio): testing the odds of male respondents reporting “a lot of difficulty” or “cannot do at all” compared to female respondents. CI: confidence interval.

**Table 4:** Age-disaggregated rates of six Washington Group functions and disability.

	Domain	5–19 years (*n* = 164)		20–49 years (*n* = 328)			50–69 years (*n* = 346)			≥ 70 years (*n* = 161)		
		*N*	% (95% CI)	*N*	% (95% CI)	Adjusted OR (95% CI)	*N*	% (95% CI)	Adjusted OR (95% CI)	*N*	% (95% CI)	Adjusted OR (95% CI)
1	Visual	18	10.9 (6.6–16.8)	30	9.1 (6.3–12.8)	0.8 (0.4–1.5)	57	16.5 (12.7–20.8)	1.6 (0.9–2.8)	59	36.7 (29.2–44.6)	4.7 (2.6–8.4)
2	Hearing	2	1.2 (0.2–4.3)	3	0.9 (0.2–2.6)	0.8 (0.1–4.6)	11	3.2 (1.6–5.6)	2.7 (0.6–12.1)	19	11.8 (7.3–17.8)	10.8 (2.5–47.3)
3	Walking	1	0.6 (0.02–3.4)	3	0.9 (0.2–2.6)	1.5 (0.2–14.8)	10	2.9 (1.4–5.3)	4.9 (0.6–38.4)	24	14.9 (9.8–21.4)	28.5 (3.8–213.7)
4	Remembering/ Concentrating	10	6.1 (3.0–10.9)	7	2.1 (0.9–4.3)	0.4 (0.1–1.0)	26	7.6 (5.0–10.8)	1.3 (0.6–2.7)	16	9.9 (5.8–15.6)	1.7 (0.7–3.9)
5	Self-care	4	2.4 (0.7–6.1)	2	0.6 (0.1–2.2)	0.2 (0.04–1.4)	3	0.9 (0.2–2.5)	0.4 (0.08–1.6)	10	6.2 (3.0–11.1)	2.6 (0.8–8.6)
6	Communicating	6	3.6 (1.4–7.8)	17	5.2 (3.1–8.2)	1.4 (0.5–3.7)	36	10.4 (7.4–14.1)	3.0 (1.3–7.4)	37	23.0 (16.7–30.3)	7.9 (3.2–19.3)
	Disability	26	15.8 (10.6–22.4)	48	14.6 (11.0–19.0)	0.9 (0.5–1.5)	103	29.8 (25.0–34.9)	2.3 (1.4–3.7)	99	61.5 (53.5–69.0)	8.5 (5.0–14.4)

Domain 1–6: number and % of respondents reporting “a lot of difficulty” or “cannot do at all”. Disability: number and % of respondents reporting “a lot of difficulty” or “cannot do at all” in at least one of the six core domains. Adjusted OR (odds ratio) reference category: Age group 5–19 years; CI: confidence interval.

**Table 5:** Cross-tabulation (affect/anxiety domain).

Level of Feelings Last Time You Felt Worried, Nervous or Anxious	How Often do You Feel Worried, Nervous or Anxious					
	Daily	Weekly	Monthly	Annually	Never	Total
Not asked	0	0	0	0	87	87
A little	15	19	18	56	0	108
In between a little and a lot	22	59	71	83	0	235
A lot	18	12	9	8	0	47
Total	55	90	98	147	87	477

Color coding: Numbers in the rows/columns with the same color form the indicator levels 1–4.

**Table 6:** Indicator (affect/anxiety domain).

Indicator Affect/Anxiety Domain	*N*	%
1	234	46.7
2	191	38.1
3	34	6.8
4 (category for having disability)	18	3.6
Excluded	24	4.8
Total	501	100

Color coding: represents the indicator levels 1–4 with the sum of the cross-tabulation numbers.

**Table 7:** Cross-tabulation (affect/depression domain).

How Depressed You Felt when Last Time You Were Depressed?	How Often do You Feel Depressed?					
	Daily	Weekly	Monthly	Annually	Never	Total
Not asked	0	0	0	0	68	68
A little	2	7	10	51	0	70
In between a little and a lot	13	26	48	86	0	170
A lot	7	17	6	9	0	39
Total	22	50	64	146	68	350

Color coding: Numbers in the rows/columns with the same color form the indicator levels 1–4.

**Table 8:** Indicator (affect/depression domain).

Indicator Affect/Depression Domain	*n*	%
1	214	42.7
2	99	19.8
3	30	6.0
4 (category for having disability)	7	1.4
Excluded	151	30.1
Total	501	100

Color coding: represents the indicator levels 1–4 with the sum of the cross-tabulation numbers.
